# Integration of the Comprehensive Physical Examination and Diagnostic Tools in Clinical Cardiology

**DOI:** 10.1002/kjm2.70074

**Published:** 2025-06-30

**Authors:** Regina Wilson, Halil Tekiner, Steven H. Yale, Eileen S. Yale

**Affiliations:** ^1^ Department of Medicine Wellington Regional Medical Center Wellington Florida USA; ^2^ Department of the History of Medicine and Ethics Erciyes University School of Medicine Kayseri Türkiye; ^3^ University of Central Florida College of Medicine Orlando Florida USA; ^4^ Nova Southeastern University Medical School, Kiran C. Patel College of Allopathic Medicine Davie Florida USA

Dear Editor,

We read with interest the case report by Chen, Chang, Wang, and Wu titled “Kussmaul's sign provoked by intracardiac metastasis of endometrial neuroendocrine tumor” [1]. While the authors offer a valuable clinical vignette, the case also provides an opportunity to revisit and clarify important historical and physiological aspects of jugular venous pulse interpretation that remain underemphasized in contemporary literature. In this letter, we aim to deepen the discussion by addressing the proper historical context of Kussmaul's sign, refining the terminology used in clinical descriptions, and elaborating on the diagnostic implications of neck pulsations and rhythm disturbances. We believe these clarifications may help readers distinguish among similar physical signs and reinforce the diagnostic value of bedside examination in complex cardiovascular presentations.

Adolf Kussmaul (1822–1902) first described in 1873 a paradoxical rise in internal jugular venous pressure during inspiration—contrary to the expected inspiratory decline—in patients with mediastino‐pericarditis with adhesion [2]. While similar findings have since been observed in restrictive cardiomyopathy, right‐sided heart failure, right ventricular infarction, pulmonary embolism, pulmonary hypertension, and cardiac tamponade, we recommend that the term “Kussmaul's sign” be reserved for the specific context Kussmaul originally described. For other conditions, the phrase “inspiratory rise in jugular venous pressure” may be more appropriate. Preserving this historical specificity is not merely semantic; terminological drift can undermine diagnostic precision, lead to confusion in clinical teaching, and dilute the interpretive value of bedside signs. Importantly, Kussmaul applied the term “paradoxical pulse” to an arterial phenomenon, not jugular venous distention [2].

Sir James Mackenzie (1853–1925) advanced the understanding of jugular venous waveforms, identifying the “a” wave as reflecting atrial contraction [3]. Paul Hamilton Wood (1907–1962) later described exaggerated “a” waves—termed “giant a waves”—and coined the term “venous Corrigan” due to their visual resemblance to the arterial Corrigan's pulse [4]. He attributed these waves to increased resistance to right ventricular filling. Giant “a” waves are typically absent when atrial contraction is ineffective (e.g., atrial fibrillation) or when right ventricular diastolic pressure is uniformly elevated (e.g., advanced right heart failure), and may be diminished in constrictive pericarditis. In the present case, although the authors did not report jugular waveform details or provide an ECG, the presence of neck pulsations suggests elevated right ventricular filling pressure and preserved atrial contraction [1].

The visible pulsation further implies that atrial fibrillation is unlikely. In sinus rhythm, the “a” wave precedes the carotid pulse; if reversed, a conduction abnormality may be present. The rhythm was likely “regularly irregular,” possibly due to sinus arrhythmia, second‐degree atrioventricular block, ectopic beats, or pulsus alternans. An ECG would have clarified this.

This case illustrates the continued value of careful physical examination alongside advanced diagnostic tools in clinical cardiology.

## Conflicts of Interest

The authors declare no conflicts of interest.

## Data Availability

Data sharing is not applicable to this article as no new data were created or analyzed in this study.
